# Avatrombopag for the treatment of thrombocytopenia induced by chemotherapy in patients with solid tumors: A multicenter, open-label, single-arm trial

**DOI:** 10.3389/fphar.2022.970978

**Published:** 2022-09-27

**Authors:** Yayun Cui, Yifu He, Changlu Hu, Congyin Tu, Jin Huang, Xiaofeng Zhu, Chunbao Zang, Kaiyang Ding, Bihong Zhan, Yufei Zhao, Liting Qian

**Affiliations:** ^1^ Department of Cancer Radiotherapy, The First Affiliated Hospital of USTC, Division of Life Sciences and Medicine, University of Science and Technology of China (Anhui Provincial Cancer Hospital), Hefei, Anhui, China; ^2^ Department of Medical Oncology, The First Affiliated Hospital of USTC, Division of Life Sciences and Medicine, University of Science and Technology of China (Anhui Provincial Cancer Hospital), Hefei, Anhui, China; ^3^ Department of Surgical Oncology, The First Affiliated Hospital of USTC, Division of Life Sciences and Medicine, University of Science and Technology of China (Anhui Provincial Cancer Hospital), Hefei, Anhui, China; ^4^ Department of Gastroenterology, The First Affiliated Hospital of USTC, Division of Life Sciences and Medicine, University of Science and Technology of China (Anhui Provincial Cancer Hospital), Hefei, Anhui, China; ^5^ Department of Hematology-Oncology, The First Affiliated Hospital of USTC, Division of Life Sciences and Medicine, University of Science and Technology of China (Anhui Provincial Cancer Hospital), Hefei, Anhui, China

**Keywords:** chemotherapy, adverse event, thrombocytopenia, receptors, thrombopoietin, agonist

## Abstract

**Objective:** To explore the effect and safety of avatrombopag for chemotherapy-induced thrombocytopenia (CIT).

**Methods:** This multicenter, open-label, single-arm trial enrolled CIT patients in eight centers from October 2020 to April 2021. The participants received avatrombopag tablets 60 mg once a day for 5–10 days. The main endpoint was the proportion of patients with platelet count ≥100×10^9^/L or increased by ≥ 50×10^9^/L or increased by ≥ 100% in the cycle after the start of treatment.

**Results:** Seventy-four participants were enrolled with a mean age of 59.8 ± 11.62.2% were males. The cumulative effective rate (any criteria) was 70.3% at 4 weeks. 42 (56.8%) achieved platelet count ≥100×10^9^/L, 44 (59.5%) increased by ≥ 50×10^9^/L, and 27 (36.5%) increase by ≥ 100% from baseline. The duration of grade III and IV platelet reduction was 4.2 ± 5.3 days. The time of PLT recovery to ≥75×10^9^/L was 9.4 ± 6.6 days. The time of PLT recovery to ≥100×10^9^/L was 10.2 ± 6.4 days. The platelet count nadir was 57.9 ± 45.3×10^9^/L. The most common adverse events were nausea (8.1%), fatigue (5.4%), and abdominal pain (1.4%). There were no cases of fever, headache, or peripheral edema.

**Conclusion:** Although it was a single-arm trial without a control group, the application of avatrombopag in patients with CIT can increase the platelet count of the patients compared with baseline. Avatrombopag is safe and tolerable.

**Clinical Trial Registration:** https://clinicaltrials.gov/ct2/show/NCT04609891?term=04609891&draw=2&rank=1, identifier [NCT04609891]

## Introduction

Chemotherapy-induced thrombocytopenia (CIT) is one of the most common complications of tumor treatment and is a common clinically hematological toxic reaction (Kuter, 2015; Weycker et al., 2019; Leader et al., 2021). The occurrence of CIT can lead to reduced chemotherapeutic drug dosage, delayed chemotherapy, and even chemotherapy termination. It may also increase the risk of bleeding in patients, endanger their life and health, affect the therapeutic effect, and increase the medical costs (Kuter, 2015; Weycker et al., 2019; Leader et al., 2021). Patients with severe CIT are at significant risk of bleeding events (Slichter et al., 2010; Stanworth et al., 2015).

The mainstay for the prophylactic or therapeutic treatment of CIT includes platelet transfusion (Stanworth et al., 2015; Schiffer et al., 2018). Platelet transfusion can decrease the rate of World Health Organization (WHO) grade ≥2 bleeding (Wandt et al., 2012; Stanworth et al., 2013), but several issues cannot be managed by transfusion, including bleeding despite platelet transfusion, delayed chemotherapy or reduced dosage, and refractoriness to platelet transfusion (Stanworth et al., 2013; Juskewitch et al., 2017; Leader et al., 2021). Therefore, platelet-stimulating growth factors, such as recombinant human interleukin 11 (rhIL-11) and recombinant human thrombopoietin (rhTPO), can be indicated to manage CIT (Leader et al., 2021). Currently, only rhTPO and rhIL-11 have been approved by the National Medical Products Administration to treat tumor-related thrombocytopenia in mainland China, but they have side effects that can impede their use. For example, rhIL-11 has a serious cardiac side effect in older adult patients, while rhTPO neutralizing antibodies may lead to a decline in long-term curative effects (Jing et al., 2018; Liu et al., 2019; Yu et al., 2021).

As a new generation of TPO receptor agonists, avatrombopag exerts its stimulating effect on megakaryocytes through binding with and activating the TPO receptors to promote the generation of platelets (Leader et al., 2021). Avatrombopag has several advantages, such as being taken orally, no hepatotoxicity, good safety, and rapid platelet elevation. Several clinical trials have confirmed the curative effect and safety of avatrombopag in chronic liver disease (CLD) patients (Xu and Cai, 2019; Poordad et al., 2020; Markham, 2021). Following the approval by the FDA and EMA, it was also approved by the NMPA in China in 2020 in adult patients with CLD who are scheduled to undergo a procedure. In addition to CLD-related thrombocytopenia, many researchers also paid attention to the curative effect of avatrombopag in CIT and immune thrombocytopenia (ITP). Benefits of avatrombopag were observed for ITP (Cheloff and Al-Samkari, 2019; Al-Samkari and Nagalla, 2022) and approved by FDA for patients who have had an insufficient response to a previous treatment. A phase three study of avatrombopag in solid tumor patients with CIT observed that composite primary outcomes (proportion of responders not requiring platelet transfusion or either a 15% or more chemotherapy dose reduction or a 4-days or more chemotherapy delay due to thrombocytopenia following study treatment until the start of the subsequent cycle) were similar between the avatrombopag and placebo groups but avatrombopag was capable to augment platelet counts. (Al-Samkari et al., 2022). Still, Al-Samkari et al. (Al-Samkari et al., 2022) raised the possibility that avatrombopag could be effective in specific populations or for different endpoints.

There is no relevant study on the application of avatrombopag in patients with CIT in China. Therefore, this study aimed to carry out a multicenter, open-label, single-arm trial to explore the effect and safety of avatrombopag for CIT. The results could provide more evidence for its clinical use.

## Methods

### Study design and population

This multicenter, open-label, single-arm trial enrolled CIT patients in eight centers from October 2020 to April 2021. This trial was approved by the Ethics Committee of the First Affiliated Hospital of USTC (Anhui Provincial *Cancer* Hospital) (L.SH. 2020 No. 71). Written informed consent was obtained from all participants. This trial was registered (ClinicalTrials.gov NCT04609891).

The main inclusion criteria were 1) 18–75 years of age, 2) malignant tumors (gynecological tumors, gastrointestinal tumors, lung cancer, gastric cancer, or head and neck tumors) by pathology or cytology and need chemotherapy, and 3) developed grade ≥2 thrombocytopenia after the last chemotherapy cycle (platelets of 10 × 10^9^–75×10^9^/L). The major exclusion criteria were 1) severe cardiovascular disease (myocardial ischemia or myocardial infarction above grade II, poorly controlled arrhythmia; according to NYHA standards, grade III to IV cardiac insufficiency, or cardiac color Doppler ultrasound examination suggests left ventricular ejection fraction (LVEF) < 50%), 2) clinically significant acute or active bleeding (such as gastrointestinal or central nervous system) within 7 days before screening, 3) received major surgery or minor surgery within 4 weeks of enrollment, or 4) history of arterial or venous thrombosis (such as myocardial ischemia, transient ischemic attack, or stroke) within 6 months before screening. Details of complete inclusion and exclusion criteria are listed in the additional materials. The detailed inclusion and exclusion criteria are listed in the Additional Material.

### Procedure

The participants received avatrombopag tablets, 20 mg/tablet, taken orally once a day (Eisai, Co., Ltd., Tokyo, Japan). All participants took avatrombopag 60 mg once a day for 5–10 days. Rescue treatment was allowed. Rescue treatment (i.e., platelet transfusion) was performed when the investigators determined that the patients needed rescue treatment. The drug withdrawal indications (at least one condition had to be met) were 1) platelet increased by 50×10^9^/L, and 2) platelet count increased to 100×10^9^/L.

### Follow-up

All participants were followed up for 4 weeks after starting the trial.

### Endpoints

The main endpoint was the proportion of patients with platelet count ≥100×10^9^/L or platelet count increased by ≥ 50×10^9^/L or platelet count increased by ≥ 100% in the cycle after the start of avatrombopag therapy.

The secondary endpoints were 1) duration of grade III and IV platelet reduction, 2) time of platelet recovery to ≥75×10^9^/L and ≥100×10^9^/L, 3) nadir platelet count, 4) proportion of patients without platelet transfusion, and 5) proportion of patients without clinical-related bleeding.

For the safety analysis, all adverse events (AEs) were recorded. AE referred to any adverse medical event that occurred after receiving the study drug, but that did not necessarily have a causal relationship with the study drug. Serious AEs (SAEs) referred to medical events that required hospitalization or prolonged hospitalization, caused disability, affected ability to work, endangered life, caused death, or led to congenital malformations during the trial.

### Statistical analysis

All participants who met the inclusion criteria and were treated with avatrombopag at least once were entered into the curative effect analytic set. All participants who had taken avatrombopag at least once and had post-medication safety records were included in the safety set.

The participants were divided into different subgroups according to whether they had bone metastasis, whether they had liver disease, tumor staging, whether they received radiotherapy, targeted therapy, or immunotherapy, the number of chemotherapy cycles, and their baseline platelet count.

SPSS 19.0 (IBM, Armonk, NY, United States) was used for statistical analysis. Continuous data obeying the normal distribution (according to the Kolmogorov-Smirnov test) were presented as means ± standard deviation and were analyzed using the *t*-test and ANOVA. Otherwise, they were presented as median (quartile) and analyzed using Wilcoxon’s rank-sum test. Categorical data were presented as n (%). All tests were two-tailed, and *p*-values <0.05 were considered statistically significant.

## Results

### Baseline characteristics

Table 1 presents the baseline characteristics of the participants. Their mean age was 59.8 ± 11.0 years. Among the 74 participants, 46 (62.2%) were male, 14 (18.9%) had lung cancer, 42 (56.7%) had digestive tumors, six (8.1%) had bone metastasis, 30 (40.5%) had metastases other than bones, 10 (13.5%) had liver disease, 12 (16.2%) was receiving radiotherapy, 22 (29.7%) was receiving targeted therapy, and 10 (13.5%) was receiving immunotherapy.

**TABLE 1:** Baseline characteristics of the participants.

Total	n = 74
Sex, n (%)	
Male	46 (62.2%)
Female	28 (37.8%)
Age mean ± SD	59.8 ± 11.0
Type of cancer, n (%)	
Lung cancer	14 (18.9%)
Esophageal cancer	9 (12.2%)
Gastric cancer	8 (10.8%)
Liver cancer	8 (10.8%)
Other digestive system tumors	17 (23%)
Urogenital system tumors	14 (18.9%)
Neuroendocrine system tumors	2 (2.7%)
Other	2 (2.7%)
*Cancer* staging	
Stage I	3 (4.5%)
Stage II	8 (11.9%)
Stage III	30 (44.8%)
Stage IV	26 (38.8%)
Osseous metastasis	6 (8.1%)
Other metastasis	30 (40.5%)
Liver disease	10 (13.5%)
Combined with radiotherapy	12 (16.2%)
Combined with targeted therapy	22 (29.7%)
Combined with immunotherapy	10 (13.5%)
Baseline platelet count (×10^9^/L)	
10–25	16 (21.6%)
25–50	33 (44.6%)
50–75	25 (33.8%)
Number of chemotherapy cycles	
<5	51 (79.7%)
≥5	13 (20.3%)

SD: standard deviation.

### Primary endpoints

The cumulative effective rate (any criteria) was 70.3% at 4 weeks (Table 2). Among the 74 participants, 42 (56.8%) achieved platelet count ≥100×10^9^/L, 44 (59.5%) achieved platelet count ≥50×10^9^/L, and 27 (36.5%) achieved platelet count increase by ≥ 100% from baseline. The results of cumulative effective rate grouping by baseline platelet count are shown in Table 3.

**TABLE 2:** Cumulative effective rate.

Follow-up	Cumulative effective rate of any criteria	Cumulative effective rate when platelet count ≥100×10^9^/L	Cumulative effective rate when increase of baseline count ≥50×10^9^/L	Cumulative effective rate when increase of baseline count ≥100%
1 week	28/74 (37.8%)	14/74 (18.9%)	16/74 (21.6%)	27/74 (36.5%)
2 weeks	47/74 (63.5%)	36/74 (48.6%)	39/74 (52.7%)	27/74 (36.5%)
3 weeks	51/74 (68.9%)	41/74 (55.4%)	43/74 (58.1%)	27/74 (36.5%)
4 weeks	52/74 (70.3%)	42/74 (56.8%)	44/74 (59.5%)	27/74 (36.5%)

**TABLE 3:** Cumulative effective rate grouping by baseline platelet count.

Follow-up	Cumulative effective rate of any criteria			Cumulative effective rate when platelet count ≥100×10^9^/L			Cumulative effective rate when increase of baseline count ≥50×10^9^/L			Cumulative effective rate when increase of baseline count ≥100%		
	Baseline Plt <25	Baseline 25 ≤ Plt<50	Baseline Plt≥50	Baseline Plt <25	Baseline 25 ≤ Plt<50	Baseline Plt≥50	Baseline Plt <25	Baseline 25 ≤ Plt<50	Baseline Plt≥50	Baseline Plt <25	Baseline 25 ≤ Plt<50	Baseline Plt≥50
1 week	10/16 (62.5%)	11/33 (33.3%)	7/25 (28%)	3/16 (18.8%)	4/33 (12.1%)	7/25 (28%)	4/16 (25%)	6/33 (18.2%)	6/25 (24%)	10/16 (62.5%)	11/33 (33.3%)	6/25 (24%)
2 weeks	13/16 (81.2%)	20/33 (60.6%)	14/25 (56%)	8/16 (50%)	14/33 (42.4%)	14/25 (56%)	11/16 (68.8%)	16/33 (48.5%)	12/25 (48%)	10/16 (62.5%)	11/33 (33.3%)	6/25 (24%)
3 weeks	13/16 (81.2%)	22/33 (66.7%)	16/25 (64%)	9/16 (56.2%)	16/33 (48.5%)	16/25 (64%)	11/16 (68.8%)	19/33 (57.6%)	13/25 (52%)	10/16 (62.5%)	11/33 (33.3%)	6/25 (24%)
4 weeks	13/16 (81.2%)	22/33 (66.7%)	17/25 (68%)	9/16 (56.2%)	16/33 (48.5%)	17/25 (68%)	11/16 (68.8%)	19/33 (57.6%)	14/25 (56%)	10/16 (62.5%)	11/33 (33.3%)	6/25 (24%)

### Secondary endpoints

The duration of grade III and IV platelet reduction was 4.2 ± 5.3 days. The time of PLT recovery to ≥75×10^9^/L was 9.4 ± 6.6 days. The time of PLT recovery to ≥100×10^9^/L was 10.2 ± 6.4 days. The platelet count nadir was 57.9 ± 45.3×10^9^/L. Among the 74 participants, 14 (18.9%) had to receive platelet transfusion as rescue treatment. Among the 74 participants, six (8.1%) had bleeding events (Table 4).

**TABLE 4:** Platelet transfusion, bleeding, and adverse events.

Index	N = 74
Platelet transfusion	14 (18.9%)
Bleeding events	6 (8.1%)
Adverse events	
Fever	0
Abdominal pain	1 (1.4%)
Nausea	6 (8.1%)
Headache	0
Fatigue	4 (5.4%)
Peripheral edema	0

### Safety

The most common AEs in this study were nausea (n = 6, 8.1%), fatigue (n = 4, 5.4%), and abdominal pain (n = 1, 1.4%). There was no fever, headache, or peripheral edema (Table 4).

### Subgroup analyses

There were no differences in the cumulative effective rate (any criteria) between participants with or without bone metastasis, other metastases, or liver disease (all *p* > 0.05) (Supplementary Table S1). There were no differences in the primary endpoint among different disease stages (all *p* > 0.05) (Supplementary Table S2). There were no differences in the cumulative effective rate (any criteria) between participants with or without radiotherapy, targeted therapy, or immunotherapy (all *p* > 0.05) (Supplementary Table S3). There were no differences in the primary endpoint between participants with <5 or ≥5 cycles of chemotherapy or among participants with different values of baseline platelets (all *p* > 0.05) (Supplementary Table S4).

## Discussion

This trial aimed to explore the effect and safety of avatrombopag for CIT. The results suggest that the application of avatrombopag in patients with CIT can significantly increase the platelet count of the patients compared with the baseline. Avatrombopag is safe and tolerable.

Wang et al. (Wang et al., 2022) suggested that rhTPO is effective and cost-effective for the management of CIT. In a meta-analysis by Zhang et al. (Zhang et al., 2017), two prevention trials of eltrombopag showed no significant effect on mortality after 33 weeks of follow-up, and there was not enough evidence to determine whether eltrombopag could reduce the incidence of at least one bleeding event (Kellum et al., 2010; Winer et al., 2015). Since that previous meta-analysis (Soff et al., 2022), some trials for the treatment of CIT have been published (Soff et al., 2022). Soff et al. (Soff et al., 2019) studied romiplostim for the treatment of CIT. The primary endpoint of that phase II trial was the platelet count recovery to ≥100×10^9^/L at 3 weeks; 14/15 (93%) of the romiplostim-treated patients achieved platelet recovery, compared with 1/8 (13%) controls. They concluded that romiplostim treatment of CIT was effective and that maintenance treatment allowed resuming chemotherapy without CIT recurrence. Al-Samkari et al. (Al-Samkari et al., 2022) investigated avatrombopag to treat CIT in patients with non-hematological cancers. Their endpoint was the composite of no need for platelet transfusion or no need for chemotherapy dose reduction by ≥ 15% or no need to delay treatments for ≥4 days; the proportion of patients achieving the primary endpoint was similar in the avatrombopag and control groups (70% vs. 73%). Gao et al. (Gao et al., 2022) reported that avatrombopag was effective in managing severe and refractory CIT. In the present trial, the results showed that the cumulative effective rate (any criteria) was 70.3% at 4 weeks, indicating that avatrombopag is effective in increasing the platelet counts in patients with CIT. These results are consistent with the effects of avatrombopag in patients with CLD and ITP (Cheloff and Al-Samkari, 2019; Al-Samkari and Nagalla, 2022).

In the present trial, among the 74 participants, six (8.1%) had bleeding events. The meta-analysis by Zhang et al. (Zhang et al., 2017) concluded that there was no sufficient evidence for the effect of eltrombopag or romiplostim on preventing bleeding events. In addition, in their trial of avatrombopag for the treatment of CIT, Al-Samkari et al. (Al-Samkari et al., 2022) did not report the bleeding events. Still, avatrombopag has been shown to prevent bleeding in CLD (Cheloff and Al-Samkari, 2019). A bleeding event in patients with CIT can be catastrophic, but not all patients with CIT will have a bleeding event, and the low occurrence of such events complicates their analysis.

It is important to note that the selection of the endpoint will affect the conclusions of a study. Indeed, the reason that the study by Al-Samkari et al. (Al-Samkari et al., 2022) was negative could be due to the selection of the composite endpoints. Indeed, they chose very clinically meaningful endpoints: delay in chemotherapy and the need for dose reduction. These endpoints can influence the course of the patient’s cancer treatments and, ultimately, their prognosis. In the present study, the duration of thrombocytopenia was selected as the primary endpoint, which is, in fact, the main action of TPO receptor agonist treatment and the reason why the treatment is given. Such an endpoint was also used in the study by Gao et al. (Gao et al., 2022) and Xu et al. (Xu et al., 2018). This endpoint is also clinically important because many clinical events and complications can occur during thrombocytopenia, such as bleeding and death and the incurring increases in medical costs (Kuter, 2015; Weycker et al., 2019; Leader et al., 2021). Furthermore, longer durations of thrombocytopenia will affect the subsequent chemotherapy treatments, either by delaying them or necessitating dose reductions, and shortening thrombocytopenia will affect the cancer treatments (Kuter, 2015; Weycker et al., 2019; Leader et al., 2021). Kuter et al. (Kuter, 2022) highlighted the importance of maintaining the chemotherapy dose intensity, and TPO receptor agonists play an important role in doing so. Therefore, even though an increase in platelet count is to be expected with TPO receptor agonists, this endpoint is important because it should affect the subsequent endpoints like dose reductions and treatment delays. Still, studies are necessary to determine whether the use of TPO receptor agonists influences patients’ prognosis, but such studies will require very large sample sizes. Based on a systematic review, Soff et al. (Soff et al., 2022) also advised conducting future trials using well-characterized bleeding and platelet thresholds.

Two TPO receptor agonists (romiplostim and eltrombopag) are currently listed in China; none were approved with indications of CIT. Previous studies reported the curative effect and safety of romiplostim and eltrombopag were compared with avatrombopag as follows. Treatment with romiplostim increased the median platelet count by more than 100% (from 54×10^9^/L to 112×10^9^/L), and 71% of patients achieved a romiplostim response (Al-Samkari et al., 2021); 79% of patients with solid tumors did not need further reduction of chemotherapy dose or treatment delay due to thrombocytopenia, and 89% of patients did not need platelet transfusion to continue treatment. In the study by Zhu et al., 28 (54.9%), 25 (50.0%), and 39 (75.0%) CIT patients received one unit of platelet transfusion in the eltrombopag, rhTPO, and control groups, respectively.

In a study of CIT in patients with solid tumors treated with rhTPO (72 patients with a platelet count of 55.9 ± 16.0 ×10^9^/L after chemotherapy), the time required for platelet recovery to ≥75×10^9^/L and ≥100×10^9^/L was 4.8 ± 3.7 and 6.9 ± 3.6 days, respectively (Dai et al., 2008). The time required in the present trial of avatrombopag was longer, perhaps because of the lower baseline platelet count than in the above study.

No new safety signal was found in the present study. Oral medications have the advantage of no reaction at the injection sites, such as pain and ecchymosis. Therefore, avatrombopag was safe and well-tolerated.

This trial has limitations. The sample size was small. There was no parallel control group. This study included patients with a variety of chemotherapy regimens, and it probably prevented the determination of the impact of avatrombopag on CIT. Due to the relatively short follow-up time, the long-term curative effect and safety could not be explored. Further studies are needed.

In conclusion, although it was a single-arm trial without a control group, the application of avatrombopag in patients with CIT can increase the platelet count of the patients compared with baseline. Avatrombopag is safe and tolerable.

## Data Availability

The original contributions presented in the study are included in the article/Supplementary Material, further inquiries can be directed to the corresponding author.
