# Supplementary material for: Avatrombopag for the treatment of thrombocytopenia induced by chemotherapy in patients with solid tumors: A multicenter, open-label, single-arm trial
Source: Front Pharmacol. 2022 Sep 27;13:970978. doi: 10.3389/fphar.2022.970978 (PMC9553127; doi:10.3389/fphar.2022.970978)
Supplement: Supplementary file 1 [file DataSheet2.docx]

**Additional Materials**

**Inclusion criteria**

1. Male or female, aged 18-75 years.
2. Diagnosed with malignant tumors by pathology or cytology and needed chemotherapy.
3. At least grade II thrombocytopenia after the last chemotherapy cycle (PLT count 10-75×10^9^/L).
4. Eastern Cooperative Oncology Group (ECOG) performance status score ≤2.
5. Life expectancy at screening was ≥12 weeks, and had to continue to receive at least two cycles of the same chemotherapy regimen at the time of screening.
6. Women of childbearing age had to use reliable contraceptive measures or had a negative pregnancy test (serum or urine) within 7 days before enrollment and were willing to use appropriate methods of contraception during the trial.
7. The subjects voluntarily and strictly abode by the requirements of the research protocol and signed the informed consent form.

**Exclusion criteria**

1. Severe cardiovascular disease: myocardial ischemia or myocardial infarction above grade II, poorly controlled arrhythmia; according to NYHA standards, grade III to IV cardiac insufficiency, or cardiac color Doppler ultrasound examination suggests left ventricular ejection fraction (LVEF) <50%.

2. Clinically significant acute or active bleeding (such as gastrointestinal or central nervous system) within 7 days before screening.

3. Received major surgery or minor surgery within 4 weeks of enrollment.

4. History of arterial or venous thrombosis (such as myocardial ischemia, transient ischemic attack, or stroke) within 6 months before screening.

5. Active infection or acute infection within 2 weeks before the first administration of the study drug.

6. Long-term bed rest, subjects with severe vascular disease.

7. History of chronic thrombocytopenia or bleeding disorders, or a history of thrombocytopenia (such as chronic liver disease or immune thrombocytopenic purpura) caused by causes other than thrombocytopenia caused by chemotherapy.

8. History of other hematological malignant tumors, such as acute lymphoblastic leukemia, acute myeloid leukemia, any myeloid malignant tumor, myelodysplastic syndrome, myeloproliferative disease, multiple myeloma, etc.

9. Factors that significantly affect the absorption of oral drugs, such as inability to swallow, chronic diarrhea, or intestinal obstruction.

10. Allergic reaction to avatrombopag or any of its excipients.

11. Participated in clinical studies of other study drugs or devices within 30 days before screening.

12. The investigator assessed that the subjects had any accompanying medical history that could impair the subject’s safety in completing the study, such as renal failure due to hemodialysis or active infection requiring intravenous antibiotics.

13. History of psychotropic drug abuse and unable to quit or have mental disorders.

14. Pregnant or breastfeeding women, and fertile patients who were unwilling or unable to take effective contraceptive measures.

15. The investigator judged that other conditions might affect the conduct of clinical research and the determination of research results.
