# Supplementary material for: Avatrombopag for the treatment of thrombocytopenia induced by chemotherapy in patients with solid tumors: A multicenter, open-label, single-arm trial
Source: Front Pharmacol. 2022 Sep 27;13:970978. doi: 10.3389/fphar.2022.970978 (PMC9553127; doi:10.3389/fphar.2022.970978)
Supplement: Supplementary file 2 [file DataSheet1.docx]

**Supplementary Table S1.** Cumulative effective rate according to the metastatic site

| Follow-up | Osseous metastasis | |  | Other metastasis | |  | Liver diseases | |  |
| --- | --- | --- | --- | --- | --- | --- | --- | --- | --- |
|  | No | Yes | P | No | Yes | P | No | Yes | P |
| 1 week | 25/68 (36.8%) | 3/6 (50.0%) | 0.661 | 16/44 (36.4%) | 12/30 (40.0%) | 0.806 | 24/64 (37.5%) | 4/10 (40.0%) | >0.999 |
| 2 weeks | 44/68 (64.7%) | 3/6 (50.0%) | 0.664 | 26/44 (59.1%) | 21/30 (70.0%) | 0.457 | 40/64 (62.5%) | 7/10 (70.0%) | 0.738 |
| 3 weeks | 48/68 (70.6%) | 3/6 (50.0%) | 0.371 | 29/44 (65.9%) | 22/30 (73.3%) | 0.603 | 44/64 (68.8%) | 7/10 (70.0%) | >0.999 |
| 4 weeks | 49/68 (72.1%) | 3/6 (50.0%) | 0.355 | 29/44 (65.9%) | 23/30 (76.7%) | 0.439 | 44/64 (68.8%) | 8/10 (80.0%) | 0.709 |

**Supplementary Table S2.** Cumulative effective rate according to staging

| Follow-up | Tumor staging | | | | P |
| --- | --- | --- | --- | --- | --- |
|  | I | II | III | IV |  |
| 1 week | 1/3 (33.3%) | 1/8 (12.5%) | 11/30 (36.7%) | 13/26 (50.0%) | 0.297 |
| 2 weeks | 2/3 (66.7%) | 3/8 (37.5%) | 19/30 (63.3%) | 20/26 (76.9%) | 0.222 |
| 3 weeks | 2/3 (66.7%) | 5/8 (62.5%) | 21/30 (70.0%) | 20/26 (76.9%) | 0.910 |
| 4 weeks | 2/3 (66.7%) | 5/8 (62.5%) | 21/30 (70.0%) | 20/26 (76.9%) | 0.901 |

**Supplementary Table S3.** Cumulative effective rates according to treatments

| Follow-up | Combined with radiotherapy | |  | Combined with targeted therapy | |  | Combined with immunotherapy | |  |
| --- | --- | --- | --- | --- | --- | --- | --- | --- | --- |
|  | No | Yes | P | No | Yes | P | No | Yes | P |
| 1 week | 21/62 (33.9%) | 7/12 (58.3%) | 0.201 | 19/52 (36.5%) | 9/22 (40.9%) | 0.797 | 26/64 (40.6%) | 2/10 (20.0%) | 0.305 |
| 2 weeks | 38/62 (61.3%) | 9/12 (75.0%) | 0.517 | 34/52 (65.4%) | 13/22 (59.1%) | 0.793 | 42/64 (65.6%) | 5/10 (50.0%) | 0.486 |
| 3 weeks | 42/62 (67.7%) | 9/12 (75.0%) | 0.735 | 38/52 (73.1%) | 13/22 (59.1%) | 0.275 | 46/64 (71.9%) | 5/10 (50.0%) | 0.276 |
| 4 weeks | 43/62 (69.4%) | 9/12 (75.0%) | 0.746 | 38/52 (73.1%) | 14/22 (63.6%) | 0.578 | 47/64 (73.4%) | 5/10 (50.0%) | 0.149 |

**Supplementary Table S4.** Cumulative effective rates according to the number of chemotherapy cycles and baseline platelets

| Follow-up time | Number of chemotherapy cycles | |  | Baseline platelet | | |  |
| --- | --- | --- | --- | --- | --- | --- | --- |
|  | <5 | ≥5 | P | 10-24.9 | 25-49.9 | 50-75 | P |
| 1 week | 21/51 (41.2%) | 4/13 (30.8%) | 0.545 | 10/16 (62.5%) | 11/33 (33.3%) | 7/25 (28.0%) | 0.067 |
| 2 weeks | 35/51 (68.6%) | 8/13 (61.5%) | 0.740 | 13/16 (81.2%) | 20/33 (60.6%) | 14/25 (56.0%) | 0.225 |
| 3 weeks | 38/51 (74.5%) | 9/13 (69.2%) | 0.723 | 13/16 (81.2%) | 22/33 (66.7%) | 16/25 (64.0%) | 0.514 |
| 4 weeks | 39/51 (76.5%) | 9/13 (69.2%) | 0.720 | 13/16 (81.2%) | 22/33 (66.7%) | 17/25 (68.0%) | 0.569 |
